# A standardised protocol for blood and cerebrospinal fluid collection and processing for biomarker research in ataxia

**DOI:** 10.1111/nan.12892

**Published:** 2023-03-29

**Authors:** Magda M. Santana, Laetitia S. Gaspar, Maria M. Pinto, Patrick Silva, Diana Adão, Dina Pereira, Joana Afonso Ribeiro, Inês Cunha, Jeannette Huebener‐Schmid, Mafalda Raposo, Ana F. Ferreira, Jennifer Faber, Sandra Kuhs, Hector Garcia‐Moreno, Kathrin Reetz, Andreas Thieme, Jon Infante, Bart P. C. van de Warrenburg, Paola Giunti, Olaf Riess, Ludger Schöls, Manuela Lima, Thomas Klockgether, Cristina Januário, Luís Pereira de Almeida, European Spinocerebellar Ataxia Type 3/Machado‐Joseph Disease Initiative Study Group, Janna Krahe, Judith van Gaalen, Cristina Gonzalez‐Robles, Zofia Fleszar, Ana Lara Pelayo‐Negro, Leire Manrique, Dagmar Timmann, Katharina M. Steiner, Ana Rosa Vieira Melo

**Affiliations:** ^1^ Center for Neuroscience and Cell Biology (CNC) University of Coimbra Coimbra Portugal; ^2^ Center for Innovative Biomedicine and Biotechnology (CIBB) University of Coimbra Coimbra Portugal; ^3^ Institute for Interdisciplinary Research University of Coimbra (IIIUC) Coimbra Portugal; ^4^ Faculty of Pharmacy University of Coimbra (FFUC) Coimbra Portugal; ^5^ Neurology Department, Child Development Centre Coimbra's Hospital and University Centre (CHUC) Coimbra Portugal; ^6^ Department of Neurology Coimbra University Hospital Center (CHUC) Coimbra Portugal; ^7^ Institute of Medical Genetics and Applied Genomics University of Tübingen Tübingen Germany; ^8^ Centre for Rare Diseases University of Tübingen Tübingen Germany; ^9^ Instituto de Biologia Molecular e Celular (IBMC), Instituto de Investigação e Inovação em Saúde (i3S) Universidade do Porto Porto Portugal; ^10^ Faculdade de Ciências e Tecnologia (FCT) Universidade dos Açores (UAc) Ponta Delgada Portugal; ^11^ DZNE, German Center for Neurodegenerative Diseases Bonn Germany; ^12^ Department of Neurology University Hospital Bonn Bonn Germany; ^13^ Ataxia Centre, Department of Clinical and Movement Neurosciences, UCL Queen Square Institute of Neurology University College London London UK; ^14^ Department of Neurogenetics, National Hospital for Neurology and Neurosurgery University College London Hospitals NHS Foundation Trust London UK; ^15^ Department of Neurology RWTH Aachen University Aachen Germany; ^16^ JARA‐BRAIN Institute Molecular Neuroscience and Neuroimaging Forschungszentrum Jülich GmbH and RWTH Aachen University Aachen Germany; ^17^ Department of Neurology Essen University Hospital Essen Germany; ^18^ Center for Translational Neuro‐ and Behavioral Sciences (C‐TNBS), Essen University Hospital University of Duisburg‐Essen Essen Germany; ^19^ Service of Neurology University Hospital Marqués de Valdecilla (IDIVAL), University of Cantabria (UC), Centro de Investigación Biomédica en Red de Enfermedades Neurodegenerativas (CIBERNED) Santander Spain; ^20^ Department of Neurology, Radboud University Medical Centre Donders Institute for Brain, Cognition and Behaviour Nijmegen The Netherlands; ^21^ Department of Neurodegenerative Diseases and Hertie‐Institute for Clinical Brain Research University of Tübingen Tübingen Germany; ^22^ German Centre for Neurodegenerative Diseases (DZNE) Tübingen Germany; ^23^ Essen University Hospital Essen Germany

**Keywords:** ataxia, biomarkers, blood, cerebrospinal fluid, neurodegenerative diseases, protocol, research, standardisation

## Abstract

The European Spinocerebellar Ataxia Type 3/Machado‐Joseph Disease Initiative (ESMI) is a consortium established with the ambition to set up the largest European longitudinal trial‐ready cohort of Spinocerebellar Ataxia Type 3/Machado‐Joseph Disease (SCA3/MJD), the most common autosomal dominantly inherited ataxia worldwide. A major focus of ESMI has been the identification of SCA3/MJD biomarkers to enable future interventional studies. As biosample collection and processing variables significantly impact the outcomes of biomarkers studies, biosampling procedures standardisation was done previously to study visit initiation. Here, we describe the ESMI consensus biosampling protocol, developed within the scope of ESMI, that ultimately might be translated to other neurodegenerative disorders, particularly ataxias, being the first step to protocol harmonisation in the field.

## Graphical abstract

In this publication, we describe the consensus biosampling protocol developed within the scope of the European Spinocerebellar Ataxia Type 3/Machado‐Joseph Disease Initiative (ESMI). This protocol was established with the aim of guiding clinical research personnel on standardized collection, processing and biobanking of blood and cerebrospinal fluid for biomarker research in ataxia. This manual has been used on an international scale and might be translated to other neurodegenerative disorders, being the first step to protocol harmonization in the field.

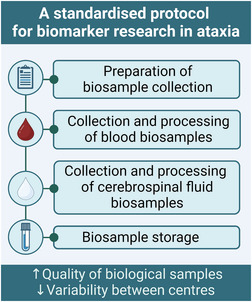

**Abbreviations**
AGI: Ataxia Global Initiative
CPT: Cell preparation tube
CSF: Cerebrospinal fluid
ESMI: European Spinocerebellar Ataxia Type 3/Machado‐Joseph Disease Initiative
EVs: Extracellular vesicles
JPND: EU Joint Programme Neurodegenerative Diseases
MJD: Machado‐Joseph Disease
MRI: Magnetic resonance imaging
MTA: Material transfer agreement
PBMCs: Peripheral blood mononuclear cells
PBS: Phosphate‐buffered saline
PolyQ: Polyglutamine tract
PPT: Plasma preparation tube
SCA3: Spinocerebellar ataxia type 3
SST: Serum separator tube

**Key points**

- The European Spinocerebellar Ataxia Type 3/Machado‐Joseph Disease Initiative (ESMI) was established with the ambition to set up the largest European longitudinal trial‐ready cohort of SCA3/MJD mutation carriers and comparable controls.
- A major focus of ESMI has also been the identification of SCA3/MJD biomarkers for future interventional studies.
- To obtain high‐quality biological samples and reduce sampling variability between centres, a biosample collection and processing manual was developed and implemented at all ESMI sites.
- The ESMI biosample collection and processing manual has been used on an international scale and might be translated to other neurodegenerative disorders.

## INTRODUCTION

Spinocerebellar Ataxia Type 3/Machado‐Joseph Disease (SCA3/MJD) is the most common autosomal dominantly inherited ataxia worldwide [1, 2]. The disease is caused by an expansion of a CAG repeat in exon 10 of the *ATXN3* gene, which is translated into an abnormally elongated polyglutamine tract (polyQ) within the ataxin‐3 protein [3, 4, 5]. This polyQ expansion confers a toxic gain‐of‐function to mutant ataxin‐3 that leads to the formation of neuronal intranuclear inclusions, neuronal dysfunction and degeneration of specific brain regions, particularly the spinal cord, the brainstem and the cerebellum [6, 7, 8]. SCA3/MJD is a progressive disease that leads to severe disability and premature death, and there is an unmet need for a therapy that can stop or delay disease progression.

Over the last years, drug‐based [9, 10, 11, 12], cellular [13, 14] and gene silencing [15, 16, 17, 18] therapies have been shown to improve disease phenotypes in several models of SCA3. However, despite the promising results of preclinical studies, the lack of reliable biomarkers is a critical factor hampering their clinical translation [19].

In this context, in 2016, the European Spinocerebellar Ataxia Type 3/Machado‐Joseph Disease Initiative (ESMI)—a consortium of institutions from Germany, the UK, the Netherlands and Portugal originally funded in the framework of the EU Joint Programme Neurodegenerative Diseases (JPND)—was established with the ambition to set up the largest European longitudinal trial‐ready cohort of SCA3/MJD mutation carriers and comparable controls. A major focus of ESMI has been the identification of SCA3/MJD biomarkers for future interventional studies. Thus, the ESMI protocol comprises not only standardised clinical assessment and magnetic resonance imaging (MRI) but also collection and storage at each centre (noncentralised biobanking) of biological specimens (whole blood, serum, plasma, peripheral blood mononuclear cells [PBMCs] and cerebrospinal fluid [CSF]).

As biosample collection and processing variables have been shown to significantly impact the outcomes of biomarkers studies [20, 21, 22], the standardisation of sample biobanking and processing, especially in the context of multicentric studies, is crucial to ensure that biosample analysis is not compromised by preanalytical factors [23, 24, 25]. Still, previous multicentric studies in the ataxia field did not have a common biosampling protocol [26, 27, 28]. To obtain high‐quality biological samples and reduce sampling variability between centres, a biosample collection and processing manual was developed and implemented at all ESMI sites.

Here, we describe the ESMI consensus biosampling protocol established with the aim of guiding clinical research personnel on standardised collection, processing and biobanking of biological samples with application on biomarkers clinical research. Although this protocol was developed within the scope of ESMI, its applicability might be translated to other ataxias being the first step to protocol harmonisation in the ataxia field. This protocol could also be envisioned as a biosampling manual for other neurodegenerative disorders.

## BIOSAMPLE COLLECTION PROTOCOL

### Preparation of biosample collection

#### Ethical considerations

The biosampling procedures described here require the informed consent of participants prior to biosample collection. Ethical committees must previously approve the study protocol that is carried out in accordance with local and international regulations.

#### Study visits

In the ESMI protocol, annual visits are scheduled and biosamples are collected at each visit. Biosampling is performed at the time of clinical evaluation, which also follows a standardised assessment. If biosamples cannot be collected at the same time as clinical evaluation, they should be collected within 1 month of the clinical visit.

#### Biosamples

Blood and CSF are collected as described in Table 1. Blood is further processed to obtain PBMCs, plasma and serum. Biosamples collected are aliquoted in cryovials (or directly stored in collection tubes) and used to isolate DNA, RNA, protein and extracellular vesicles (EVs).

**TABLE 1:** Biosampling description. Type of biosample and required collection tubes and blood volumes for each purpose.

Sample		Collection tube		Number × volume	Purpose	Baseline visit	Follow‐up visit(s)
Blood	Whole blood	#1 #2	PAXgene RNA	2 × 2.5 mL	RNA (RNAseq/qPCR)	X	X
	PBMCs	#3 #4	Cell preparation tube (CPT)	2 × 8.0 mL	Protein RNA	X	X
	Serum	#5 #6 #7	Serum separator tube (SST)	3 × 8.5 mL	EVs Protein	X	X
	Plasma	#8 #9	Plasma preparation tube (PPT)	1 × 8.5 mL	EVs Protein	X	X
	Whole blood	#10	EDTA tube	1 × 4.0 mL	DNA	X	Optional
Cerebrospinal fluid		#11	Polypropylene tube	1 × 15 mL	RNA Protein EVs	X	X

Abbreviation: PBMC, peripheral blood mononuclear cells.

#### Collection tubes

Collection tubes were carefully selected according to the type of biosamples being collected. Tubes should not be supplemented or replaced by tubes from other suppliers unless approval to do so is granted by the Biosample Coordination Site. A description of the different types of collection tubes and appropriate storage conditions, before sample collection, is presented in Table 2 (more detailed information about each tube can be obtained from the supplier).

**TABLE 2:** Collection tubes used for biosampling. Description of the collection tube, reference, cap colour, additives and storage conditions required for each type of biosample.

Sample			Collection tube	Reference	Cap colour	Additives	Storage
Blood	Whole blood	#1 #2	PAXgene RNA	BD #762165	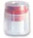	RNA stabilising agent	18–25°C
	PBMC	#3 #4	Cell Preparation Tube (CPT)	BD #362780	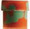	Sodium Heparin/Ficoll	18–25°C
	Serum	#5 #6 #7	Serum Separator Tube (SST)	BD #367953	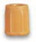	Clot Activator/Gel	4–25°C
	Plasma	#8 #9	Plasma Preparation Tube (PPT)	BD #362799	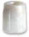	K_2_EDTA/Gel	4–25°C
	Whole blood	#10	EDTA Tube	BD #367839	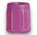	K_2_EDTA	4–25°C
Cerebrospinal fluid		#11	Polypropylene Tube	BD #352096 (or equivalent)	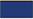	No additives	‐

Abbreviation: PBMC, peripheral blood mononuclear cells.

#### Equipment and materials

In order to collect and process samples consistently across all centres and to ensure the highest sample quality, sites must have access to the following equipment:

- Swing‐out rotor‐type centrifuge at room temperature;
−20°C freezer;
−80°C freezer.

Each centre is responsible to ensure that collection blood kits are compatible with the tubes described in Table 2. A list of the material necessary for blood collection and processing can be found in the supporting information Annex I.

#### Biosample identification

##### Collection tubes labelling

Each collection tube should be unequivocally labelled prior to blood collection. Each centre should use its local labelling system and codes, but efforts must be made to evade handwriting in labelling to avoid any source of misunderstanding. Labels should be printed and pasted on the tubes. The following recommendations should be followed when labelling the tubes:

- Confirm patient identity before collecting the blood;
- Place the label vertically on the tube;
- Avoid misaligned labelling;
- Ensure the label has completely adhered to the tube.

##### Aliquot labelling

After blood sample processing, cryovials will be used to store PBMCs and aliquots of plasma/serum/whole blood. Each cryovial should be unequivocally labelled before blood processing. Each centre will use their local labelling system, but efforts should be done to avoid handwriting in the labelling to avoid misspelling words.

#### Common biosample code

Samples to be exchanged between centres need to be identified by a common biosample code, generated as follows: patient ID/no. of visit, no. of original blood tube and no. of the aliquot. For example, a biosample labelled with the code ‘3475490/172’ corresponds to the aliquot no. 2 from tube #7 (i.e. serum), collected at the baseline visit (1), from patient 3475490. For tubes #5 and #6, PBMCs will correspond to aliquot 0. A representative table with the code generation can be found in the supporting information Annex II.

Every time a biosample is shared between centres, it must be accompanied by a biosample sheet (supporting information Annex III) that matches the local biosample code (if used) to this common biosample code.

#### Records

To have a written record of each biosample collection procedure, a biosample identification form (supporting information Annexes IV and V) must be filled out every time biosampling is collected.

The biosample identification form contains the following information:

- Research centre;
- Visit number;
- Date of the collection;
- Hour of the collection;
- Identification number of the sample/patient;
- Gender and age of the patient;
- Number of CAG repeats;
- Disease onset;
- SARA score;
- Time of the last meal;
- Number of tubes collected;
- Any occurrence out of ordinary during collection and processing.

Additionally, the back of the sample identification form might be used to add any relevant information/observations related to sample collection and processing.

### Collection and processing of blood biosamples

#### Participants preparation

Efforts must be made to ensure the procedure is as easy and painless as possible. Technicians must remain calm and project an attitude of competence and confidence even when faced with the most nervous or inquiring participants. Approximately 67 mL of blood is collected from each participant. The technician should inform any participant who is concerned about the volume of blood collected in this protocol that the total amount drawn is almost seven times less than the volume usually collected in a blood donation.

Note: this biosampling protocol was developed for adult participants, as SCA3 is an adult‐onset disease.

#### Time for blood collection

Blood samples should be collected during the morning between 8:00 and 12:00, preferably fasting. The time of blood collection and the last meal must be recorded in the biosample identification form.

#### Order of tube collection

Technicians should be previously familiarised with the different tubes. The biosample collection protocol, and a blood collection tray with the tubes in the correct order, should be prepared in advance.

To guarantee the collection of all the different biosample types, tubes should be filled with the recommended volumes of blood in the following order:

#1. PAX gene RNA tube 2.5 mL

#3. Cell preparation tube (CPT) 8.0 mL

#5. Serum separator tube (SST) 8.5 mL

#8. Plasma preparation tube (PPT) 8.5 mL

#10. EDTA tube 4.0 mL

#9. PPT 8.5 mL

#6. SST 8.5 mL

#2. PAX gene RNA tube 2.5 mL

#4. CPT 8.0 mL

#7. SST 8.5 mL

A representative picture of a tray with the blood collection tubes, in this specific order, can be found in the supporting information Annex VI.

The number of collected tubes must be registered in the blood biosample identification form (fill out the checklist with X for each tube collected).

#### Venipuncture and blood collection

Blood collection should be performed according to recommended guidelines for standard venipuncture. General recommendations should be followed, such as the following:

- Assess participant disposition.
- Confirm participant identity.
- Place the donor's arm in a downward position.
- Hold collection tubes in a vertical position, below the donor's arm during blood collection.
  [CRITICAL STEP]: Make sure tube additives do not touch the stopper or end of the needle during venipuncture.
- Release the tourniquet as soon as blood starts to flow into the tube.
- Make sure the tubes are totally filled.
- Immediately invert the tube, in accordance with the number of inversions recommended for each tube type (see Table 3).
  [CRITICAL STEP]: Tubes must be immediately mixed by complete inversion according to Table 3.
- Tubes should then be stored upright, at room temperature, until processing.

**TABLE 3:** Number of tube inversions after blood collection.

Number	Collection tube	Cap	Number of inversions
#1 #2	PAXgene tube	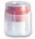	8–10 times
#3 #4	Cell preparation tube (CPT)	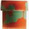	8–10 times
#5 #6 #7	Serum separator tube (SST)	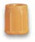	5 times
#8 #9	Plasma preparation tube (PPT)	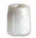	8–10 times
#10	EDTA tube	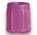	8–10 times

A representative picture of a workflow of the venipuncture procedure can be seen in the supporting information Annex VII.

#### Processing of blood biosamples

[CRITICAL STEP]: Blood processing must start as soon as possible and within a timeframe of 2 h after blood collection to ensure high‐quality samples. If sample processing starts after a period of 2 h of collection, it must be reported in the sample identification form.

Specific instructions for blood processing for each blood collection tube are described below.

***PAXgene Tube (#1 and #2)***

- Incubate tubes upright, at room temperature, for at least 2 h and no more than 6 h.
- Freeze tubes at −20°C for 24 to 72 h.
- Store tubes at −80°C.

**FIGURE 1:**
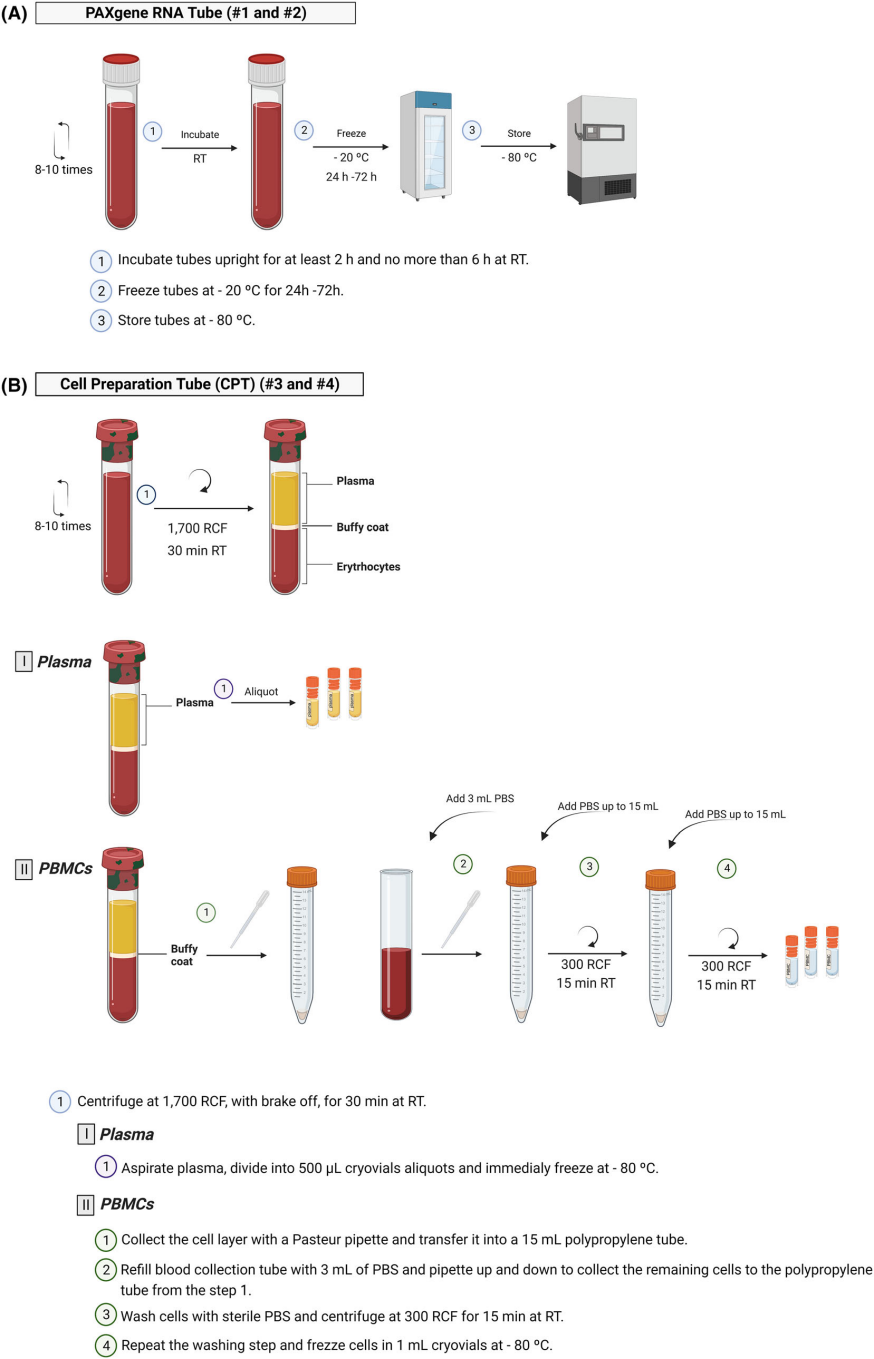
Protocol for PAXgene tube and cell preparation tube processing. Workflow summarising the protocol for RNA PAXgene tube (A) and cell preparation tube (B) processing to obtain whole blood and peripheral blood mononuclear cells (PBMCs) and plasma for biomarker studies, respectively. This picture was drawn specifically for this publication and is not included in ESMI biosampling manual. Created with BioRender.com (agreement number: JK24YGCI87).

A representative workflow of PAXgene blood processing is shown in Figure 1A.

***CPT (#3 and #4)***

- Centrifuge tubes at room temperature in a balanced, swing‐out rotor‐type, for 30 min at 1700 RCF, with the brake off.
- After centrifugation, plasma will become evident at the top layer, while PBMCs and platelets will appear as a whitish ring just under the plasma layer.

- For plasma collection

1. Aspirate plasma without disturbing the cell ring and divide it into 500 μL cryovial aliquots.
2. Immediately freeze at −80°C.
  Note: Less than 500 μL aliquots should be saved to the extent that local conditions allow.

- For PBMCs collection

1. Collect the cell layer with a sterile Pasteur pipette and transfer it to a 15 mL polypropylene tube.
2. Refill the blood collection tube with 3 mL PBS and pipette up and down to collect the remaining cells.
3. Transfer PBS/PBMCs with a sterile Pasteur Pipette to the polypropylene tube from step 1.
4. Wash cells with sterile phosphate‐buffered saline (PBS), without magnesium and calcium, by adding PBS up to the 15 mL mark.
5. Centrifuge for 15 min at 300 RCF, at room temperature, with the brake on.
6. Discard the supernatant.
7. Repeat the washing step: resuspend the PBMCs pellet in 3 mL of PBS, add PBS up to the 15 mL mark and centrifuge for 15 min, at room temperature, at 300 RCF with the brake on.
8. At this step, choose one of the options:

Option 1 (if a microcentrifuge is available; to optimise storage space):

- Discard the supernatant;
- Resuspend the PBMCs pellet in 1 mL of PBS;
- Transfer to a cryovial and centrifuge at 300 RCF, at room temperature, with the brake on;
- Discard the supernatant removing all the PBS from the cell pellet;
- Freeze the cryovials with the cell pellet at −80°C.

Option 2:

- Discard the supernatant removing all the PBS from the cell pellet;
- Freeze the 15 mL polypropylene tube with the cell pellet at −80°C.

A representative workflow of CPT blood processing is shown in Figure 1B.

***SST (#5, #6 and #7)***

- Allow blood to clot in a vertical position for a minimum of 30 min and no more than 2 h.
- Centrifuge tubes in a balanced, swing‐out rotor‐type centrifuge, at room temperature, at 1100 RCF for 10 min, with the brake off.
- Remove the BD Hemogard™ Closure. Aspirate the serum layer with a 10 mL syringe, using a 16G needle. Alternatively, a sterile Pasteur pipette can be used to transfer serum into the 10 mL syringe.
- Replace the needle with a 0.8 μm filter and push the serum through the filter drop by drop into a 15 mL polypropylene tube.

Note: When aspirating into the syringe/Pasteur pipette, be sure not to disturb the red cell layer/buffy coat with the tip of the needle.

- Divide serum into 500 μL cryovial aliquots and freeze immediately at −80°C.

Note: Less than 500 μL aliquots should be saved to the extent that local conditions allow.

**FIGURE 2:**
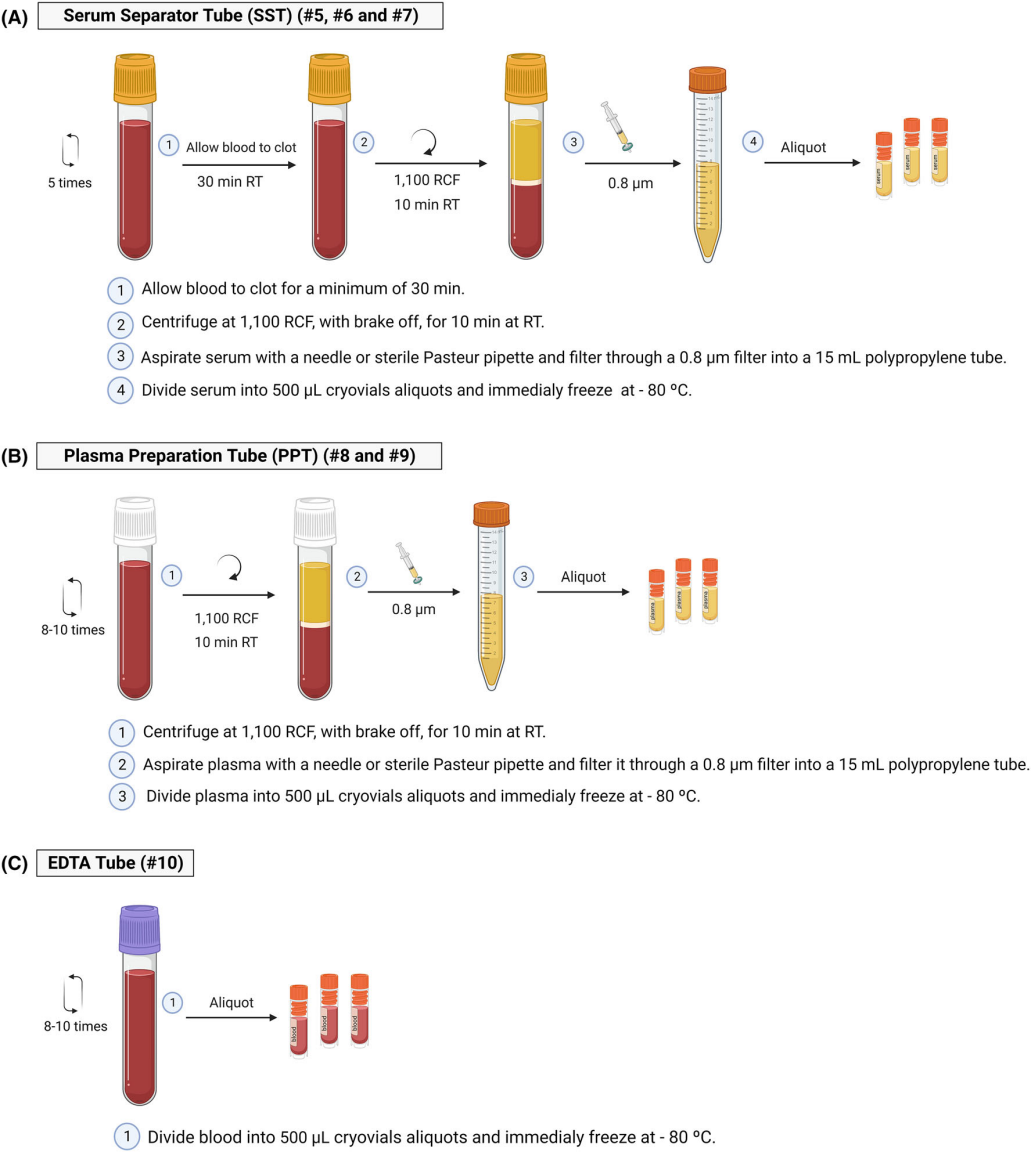
Protocol for serum separator tube, plasma preparation tube and EDTA tube processing. Workflow summarising the protocol for serum separator tube (A), plasma preparation tube (B) and EDTA tube (C) processing to obtain serum, plasma and whole blood for biomarker studies, respectively. This picture was drawn specifically for this publication and is not included in European Spinocerebellar Ataxia Type 3/Machado‐Joseph Disease Initiative (ESMI) biosampling manual. Created with BioRender.com (agreement number: DF250CCVQ0).

A representative workflow of SST blood processing is shown in Figure 2A.

***PPT (#8 and #9)***

- Centrifuge tube in a balanced, swing‐out rotor‐type centrifuge at room temperature at 1100 RCF for 10 min, with the brake off.
- Remove the BD Hemogard™ Closure.
- Aspirate the plasma layer into a 10 mL syringe using a 16G needle. Alternatively, a sterile Pasteur pipette can be used to transfer plasma into the 10 mL syringe.
- Replace the needle with a 0.8 μm filter and push the plasma through the filter drop by drop into a 15 mL polypropylene tube.

Note: When aspirating into the syringe/Pasteur pipette, be sure NOT to disturb the red cell layer/buffy coat with the tip of the needle.

- Divide plasma into 500 μL cryovial aliquots and freeze immediately at −80°C.

Note: Less than 500 μL aliquots should be saved to the extent that local conditions allow.

A representative workflow of PPT blood processing is presented in Figure 2B.

***EDTA Tube (#10)***

- Divide blood into 500 μL cryovial aliquots.

Note: Less than 500 μL aliquots should be saved to the extent that local conditions allow.

- Freeze directly at −80°C.

A representative workflow of EDTA blood processing is shown in Figure 2C. An overview table for blood collection and processing can be found in the supporting information Annex VIII.

### Collection and processing of CSF biosamples

#### Participants preparation

Each participant must be informed about the importance of the procedure in the context of the study. Potential complications and risks should be discussed with the clinicians. Clinicians and technicians must make efforts to optimise patient comfort and minimise risks of adverse events. CSF should be collected in the morning, preferably fasting. Record the time of the last meal on the CSF Sample Identification Form (supporting information Annex V).

#### Lumbar puncture and CSF collection

The lumbar puncture should be performed according to each centre's recommended standard operating procedure, by trained personnel.

The following recommendations should be followed:

- Assess participant disposition;
- Confirm participant identity;
- Ensure antiseptic cleansing and anaesthesia;
- In case of bleeding at the puncture site, discard the first 1 mL of CSF and record the event on the CSF biosample identification form;
- Collect between 12 and 15 mL of CSF in a polypropylene tube;
- Mix the CSF gently by turning the tube upside down three to four times (cap on).

#### Processing of CSF biosamples

[CRITICAL STEP]: The CSF processing must start as soon as possible and within a timeframe of 2 h after CSF collection to ensure the high quality of samples. If sample processing starts after a period of 2 h, it must be reported in the biosample identification form.

Specific instructions for CSF processing are the following:

- Centrifuge the tube in a balanced, swing‐out rotor‐type centrifuge, at room temperature, for 10 min at 1100 RCF.
- Aspirate the CSF and divide the fluid into 500 μL cryovial aliquots.

Note: Less than 500 μL aliquots should be saved to the extent that local conditions allow.

- Freeze immediately at −80°C.

#### Reporting

The biosample identification form (supporting information Annexes IV and V) should be filled immediately after blood/CSF collection and processing. Any deviation from this protocol or any occurrence during collection and processing (e.g. haemolysis during collection, timeframe for blood processing not respected and sample contamination) must be reported in the biosample identification form.

### Storage of biosamples

Storage of biomaterials should be done locally in appropriate cooling devices at −80°C, after appropriate processing.

## FINAL REMARKS

The collection of biological samples is an integral part of clinical research studies. Biological samples are, however, subject to different collecting, processing and storage conditions that can significantly alter their molecular composition, affecting experimental results and the ability to reproduce accurate scientific data [29, 30, 31]. Therefore, the development of guidelines or standardised protocols for biosample collection and processing is extremely important to ensure that variation in measurement values reflects true biological differences between samples rather than differences in biosampling procedures.

Taking this into consideration, we present here the standardised protocol of the ESMI consortium for the collection, processing and storage of blood and CSF samples from SCA3/MJD patients and control subjects.

The ESMI project was successfully kicked off with a meeting in Bonn (Germany) in May 2016. The first version of this manual was written in June 2016 and implemented over a trial period of 6 months in 6 research centres. It is important to highlight that the type and number of collected tubes, and processing procedures, were defined in accordance with the project aims. These included (1) transcriptomic profiling of whole blood to identify deregulated transcripts, to detect noncoding RNAs and alternative splicing; (2) quantification of ataxin‐3 and other protein candidates (e.g. Neurofilaments, GFAP, Tau) in serum, plasma, CSF and/or PBMCs; and (3) transcriptomic analysis of small RNAs in EVs isolated from plasma/serum. Accordingly, PAXgene tubes were selected for transcriptomic RNA studies [32, 33], CPT tubes for PBMCs isolation [34, 35] and SST and PPT tubes for serum and plasma isolation, respectively, for protein analysis and for EVs isolation [36]. A K2EDTA tube was also included to collect whole blood for genotyping, including the number of CAG repeats in the *ATXN3* gene. A filtration step was included in plasma and serum processing as required for EV isolation [36].

In December 2016, a second version of the protocol was written, implemented and followed in a total of 11 research centres (six consortium partners and five associated recruitment centres). This version accounted for specificities of individual research centres, namely, related to ethical approvals, equipment availability, time to initiate processing of biospecimens and storage temperatures. In brief, two research centres were unable to collect all the blood collection tubes due to ethics constraints and the unavailability of appropriate centrifuges at the local site of blood collection. The time defined to initiate the processing of biological samples and storage temperatures was adjusted to assure that all research centres could comply with it.

During a 7‐year timespan, biosamples were collected and stored in local biobanks of participating research centres of the consortium. In total, about 21,952 blood aliquots were collected during study visits, of which 8354 were collected at baseline, 6166 at the first‐year follow‐up visit, 4253 at the second‐year follow‐up visit, 1943 at the third‐year follow‐up visit, 917 at the fourth‐year follow‐up visit and 319 at the fifth‐year follow‐up visit (Table 4). Blood samples are available from 440 ESMI subjects (339 SCA3 mutation carriers, 7 at‐risk subjects, not diagnostically tested yet and 94 healthy controls), which corresponded to 80.4% of all enrolled subjects at baseline. At the annual follow‐up visits, blood samples were provided by around 81.7% of the examined subjects.

**TABLE 4:** Biosamples collected during ESMI study visits.

	Baseline		Follow‐up 1		Follow‐up 2		Follow‐up 3		Follow‐up 4		Follow‐up 5		Total	
	Tubes	Aliquots^a^	Tubes	Aliquots^a^	Tubes	Aliquots^a^	Tubes	Aliquots^a^	Tubes	Aliquots^a^	Tubes	Aliquots^a^	Tubes	Aliquots^a^
RNA PAXgene	1478	NA	1018	NA	512	NA	174	NA	68	NA	19	NA	3269	NA
PBMC CPT	670	670	494	494	310	310	130	130	61	61	23	23	1688	1688
Plasma CPT	670	2680	494	1976	310	1240	130	520	61	244	23	92	1688	6752
Serum STT	794	2382	617	1851	457	1371	215	645	102	306	33	99	2218	6654
Plasma PPT	457	1371	371	1113	280	840	149	447	68	204	23	69	1348	4044
Whole blood EDTA	417	1251	244	732	164	492	67	201	34	102	12	36	938	2814

Abbreviations: CPT, cell preparation tube; ESMI, European Spinocerebellar Ataxia Type 3/Machado‐Joseph Disease Initiative; NA, not applicable; PBMC, peripheral blood mononuclear cell; SST, serum separator tube; PPT, plasma preparation tube.

^a^ Number of aliquots is estimated: cell preparation tube (CPT): one aliquot of peripheral blood mononuclear cells (PBMCs) and approximately four aliquots of plasma, per tube; serum separator tube (SST): approximately three aliquots of serum, per tube; plasma preparation tube (PPT): approximately three aliquots of plasma per tube; EDTA tube: approximately three aliquots of whole blood per tube.

Additionally, 59 samples of CSF were collected during ESMI study visits. CSF is extremely valuable for ataxia‐related research questions and will play a key role in future clinical trials. However, since its collection implies a lumbar puncture, only a relatively small proportion of ESMI subjects agreed to donate CSF. Patients' awareness of the importance of CSF collection for SCA biomarker studies and the development of therapeutic strategies are needed.

After signing a material transfer agreement (MTA), samples were transferred between sites for biomarker studies approved by the ESMI Executive Board. The standardisation of these procedures has been extremely valuable and already allowed us to develop an assay for ataxin‐3 quantification [33] and to establish neurofilaments as biomarkers for MJD/SCA3 [37, 38, 39]. Biosamples have since been used in many other studies that are still ongoing and profit from the adherence to this protocol.

In 2020, a third version of the ESMI manual (the one presented in this manuscript) was implemented to maintain the collection and processing of biological samples during clinical visits, with fewer resources. In this last version of the manual, the quantity and type of tubes used were revised. The number of PAXgene tubes was decreased from four to two, and the number of serum and plasma tubes was increased from two to three and one to two, respectively. Despite their extensive use in transcriptomic biomarker studies [40, 41, 42], PAXgene tubes are expensive, which restricts their use. Conversely, PPT and SST tubes are more cost‐affordable and allow serum and plasma collection, two of the most clinically relevant and used biofluids. In addition, the aliquot volume of biosamples was also decreased from 1000 to 500 μL. Nowadays, analytical technologies are more sensitive, requiring lower volumes of input material. The storage of small volumes is thus preferred to avoid repeated freeze/thaw cycles of larger aliquots [29]. Aliquots of smaller volumes also increase biomaterials availability for a larger number of studies. As lack of storage space was one of the major hurdles reported by research centres, the presented version also included the optional suggestion to discard low‐volume aliquots of samples. In this last version, aligned with what was referred to above, partners were requested to store all collected materials (even small volumes), to the extent that local conditions allow.

Biomarker research is a continuous process that begins with the discovery phase followed by a validation process, ultimately leading to implementation in a clinical setting [43]. This is an extensive and demanding process where collaborations usually take place. To be able to move from research to clinical application, standardisation of methodologies is mandatory to allow replication of studies and comparison of results at a global scale.

Until very recently, global ataxia research was performed independently by research centres, with independent infrastructures and procedures. In 2018, a worldwide initiative for clinical research in ataxia, the Ataxia Global Initiative (AGI) (https://ataxia-global-initiative.net), was initiated with the aim of establishing consensus on standards for clinical trials on SCA3/MJD and other ataxias, among academic institutions, industry companies and regulatory authorities. The standard protocols for clinical assessment, MRI and sampling of biofluids developed for ESMI have been the basis of consensus protocols currently established by the respective AGI working group.

We expect that the standardisation of procedures in the ataxia field will help to get further insights into disease pathophysiology and to establish more reliable studies for biomarker research and development, ultimately contributing to the treatment of SCA3/MJD and other ataxias.

## AUTHOR CONTRIBUTIONS

**Magda M Santana:** conceptualization, methodology, protocol design, experimental validation of the protocol in the laboratory setting and writing—original draft. **Laetitia S Gaspar:** methodology, experimental validation of the protocol in the laboratory setting, and writing—original draft. **Maria M Pinto:** figure preparation and writing—original draft. **Patrick Silva:** experimental validation of the protocol in the laboratory setting and writing—original draft. **Diana Adão:** writing—review and editing. **Dina Pereira:** experimental validation of the protocol in the laboratory setting and writing—review and editing**. Joana Afonso Ribeiro:** experimental validation of the protocol in the clinical setting and writing—review and editing. **Inês Cunha:** experimental validation of the protocol in the clinical setting and writing—review and editing. **Jeannette Huebener‐Schmid:** experimental validation of the protocol and writing—review and editing. **Mafalda Raposo:** experimental validation of the protocol and writing—review and editing. **Ana F Ferreira:** experimental validation of the protocol and writing—review and editing. **Jennifer Faber:** writing—review and editing. **Sandra Kuhs:** experimental validation of the protocol. **Hector Garcia‐Moreno:** experimental validation of the protocol and writing—review and editing. **Kathrin Reetz:** writing—review and editing. **Andreas Thieme:** writing—review and editing**. Jon Infante:** writing—review and editing. **Bart P.C. van de Warrenburg:** writing—review and editing. **Paola Giunti:** writing—review and editing. **Olaf Riess:** writing—review and editing. **Ludger Schöls:** writing—review and editing**. Manuela Lima:** writing—review and editing. **Thomas Klockgether:** project coordination and writing—review and editing. **Cristina Januário:** experimental validation of the protocol in the clinical setting and writing—review and editing. **Luís Pereira de Almeida:** project coordination and writing—review and editing.

## CONFLICT OF INTEREST STATEMENT

JAR is a participant of advisory boards for Biogen, Roche and Novartis and received speaking fees for Biogen. JF received funding as a fellow of the Hertie Network of Excellence in Clinical Neuroscience; KR has received grants from the German Federal Ministry of Education and Research (BMBF 01GQ1402, 01DN18022), the German Research Foundation (IRTG 2150), Alzheimer Forschung Initiative e.V. (NL‐18002CB), Friedreich's Ataxia Research Alliance (FARA) and honoraria for presentations or advisory boards from Biogen and Roche. BvdW receives research support from ZonMW, Hersenstichting, Gossweiler Foundation and Radboud University Medical Center; he has served on the scientific advisory board of uniQure and receives royalties from BSL‐Springer Nature; PG has received grants and personal fees from Vico Therapeutics, personal fees from Triplet Therapeutics, grants and personal fees from Reata Pharmaceutical and grants from Wave. OR received institutional grants from Illumina for the implementation of genome analysis into diagnostics and to study the host contribution to COVID19 disease severity. TK receives/has received research support from the Deutsche Forschungsgemeinschaft (DFG), the Bundesministerium für Bildung und Forschung (BMBF), the Bundesministerium für Gesundheit (BMG), the Robert Bosch Foundation, the European Union (EU) and the National Institutes of Health (NIH). He has received consulting fees from Biogen, Biohaven, Roche, UCB, Uniqure and Vico Therapeutics. He has received a speaker honorarium from Novartis and Bayer. LPA research group has private funding from PTC Therapeutics, Uniqure, Wave life Sciences, Blade Therapeutics and Hoffmann‐La Roche AG outside the submitted work. MMS, LSG, MMP, PS, DA, DP, IC, JHS, MR, AFF, JF, SK, HGM, AT, JI, LS, ML and CJ have no disclosures.

## FUNDING INFORMATION

This project was supported by the EU Joint Programme—Neurodegenerative Disease Research (JPND) through the following funding organisations under the aegis of JPND: Portugal, Foundation for Science and Technology (FCT, grant number JPCOFUND/0001/2015) and Regional Fund for Science and Technology of the Azores; Germany, Federal Ministry of Education and Research (BMBF; funding codes 01ED1602A/B); Netherlands, The Netherlands Organisation for Health Research and Development; United Kingdom, Medical Research Council. This project has received funding from the European Union's Horizon 2020 research and innovation programme under grant agreement number 643417. In addition, support has been received by the ERDF through the Regional Operational Program Center 2020, Competitiveness Factors Operational Program (COMPETE 2020, POCI) and National Funds through FCT [BrainHealth2020 (CENTRO‐01‐0145‐FEDER‐000008), UID/NEU/04539/2019–2021, BDforMJD (CENTRO‐01‐0145‐FEDER‐181240), ViraVector (CENTRO‐01‐0145‐FEDER‐022095), SpreadSilencing (POCI‐01‐0145‐ FEDER‐029716)], and by the National Ataxia Foundation (USA), the American Portuguese Biomedical Research Fund (APBRF) and the Richard Chin and Lily Lock Machado‐Joseph Disease Research Fund. MR (CEECIND/03018/2018), AFF (SFRH/BD/121101/2016), LG (PD/BD/135497/2018) and PS (SFRH/BD/148451/2019) are supported by FCT. PG is supported by the National Institute for Health Research University College London Hospitals Biomedical Research Centre UCLH. PG receives also support from the North Thames CRN. PG and HGM, work at University College London Hospitals/University College London, which receives a proportion of funding from the Department of Health's National Institute for Health Research Biomedical Research Centre's funding scheme. PG received funding from CureSCA3 in support of HGM work.

### ETHICS STATEMENT

No ethical approval is needed.

### PEER REVIEW

The peer review history for this article is available at https://publons.com/publon/10.1111/nan.12892.

## Supporting information


**Data S2.** Supporting Information

## Data Availability

Data sharing is not applicable to this article as no new data were created or analysed in this study.

## APPENDIX A ESMI CO‐INVESTIGATORS

**Table:**

Name	Location	Role	Contribution
Janna Krahe	Department of Neurology, RWTH Aachen University	Study coordinator	Study support, technical assistance
Judith van Gaalen	Radboud University Medical Center	Site investigator	Protocol implementation at the study site
Cristina Gonzalez‐Robles, MD	Ataxia Centre, Department of Clinical and Movement Neurosciences, UCL Queen Square Institute of Neurology, University College London, London, UK	Site investigator	Protocol implementation at the study site, data uploading
Zofia Fleszar, MD	Ataxia Centre, Department of Clinical and Movement Neurosciences, UCL Queen Square Institute of Neurology, University College London, London, UK	Site investigator	Protocol implementation at the study site
Ana Lara Pelayo‐Negro, MD, PhD	University Hospital Marqués de Valdecilla‐IDIVAL, Santander, Spain	Site investigator	Protocol implementation at the study site
Leire Manrique, MD	University Hospital Marqués de Valdecilla‐IDIVAL, Santander, Spain	Site investigator	Protocol implementation at the study site
Dagmar Timmann	Essen University Hospital, Germany	Site investigator	Protocol implementation at the study site
Katharina M. Steiner	Essen University Hospital, Germany	Site investigator	Protocol implementation at the study site
Ana Rosa Vieira Melo	Universidade dos Açores, Ponta Delgada, Portugal	Site investigator	Protocol implementation at the study site
