# Supplementary material for: A standardised protocol for blood and cerebrospinal fluid collection and processing for biomarker research in ataxia
Source: Neuropathol Appl Neurobiol. 2023 Mar 29;49(2):e12892. doi: 10.1111/nan.12892 (PMC10947376; doi:10.1111/nan.12892)
Supplement: Supplementary file 1 — Data S2. Supporting Information [file NAN-49-0-s001.docx]

**A standardized protocol for blood and cerebrospinal fluid collection and processing for biomarker research in ataxia**

**- Supplementary Material -**

**ANNEX I – Material and reagents for blood collection and processing**

**Blood collection**

- 1 x 21G butterfly needle
- 1 x Vacutainer needle holder
- 1 x Latex gloves
- 1 x Tourniquet
- Alcohol wipes
- Cotton wool
- Small plasters
- 1 x Sharps bin for used needle or needle/holder combination
- Sample tubes:

2 x PAXgene RNA Tube 2.5 mL (BD #762165)

2 x Cell Preparation Tube 8.0 mL (BD #362780)

3 x Serum Separator Tube 8.5 mL (BD #367953)

2 x Plasma Preparation Tube 8.5 mL (BD #362799)

1 x EDTA Tube 4.0 mL (BD #367839)

- Tube labels
- Sample Identification Form

**Blood processing**

- Sterile Pasteur pipettes
- 5 ml pipettes
- 10 mL syringes
- 16 G sterile needles (optional, sterile Pasteur pipettes can be used instead)
- 0.8-micron filters (Merck Millipore #SLAA033SS)
- 15 mL conical sterile polypropylene tubes
- 1 mL working volume cryovials
- Sterile Phosphate Buffered Saline, without magnesium and calcium
- 1 mL micropipettes
- 1 mL micropipette tips with filter

**ANNEX II – General ESMI biosample codes**

***Code generation:***

**ESMI Patient ID / Nº of visit; Nº of original blood tube; Nº of the aliquot**

***Note:*** For CPT, PBMC will correspond to aliquot 0.

 ***ESMI PATIENT ID:* 3475490** *(hypothetical patient)*

**ANNEX III – Biosample sheet**

**
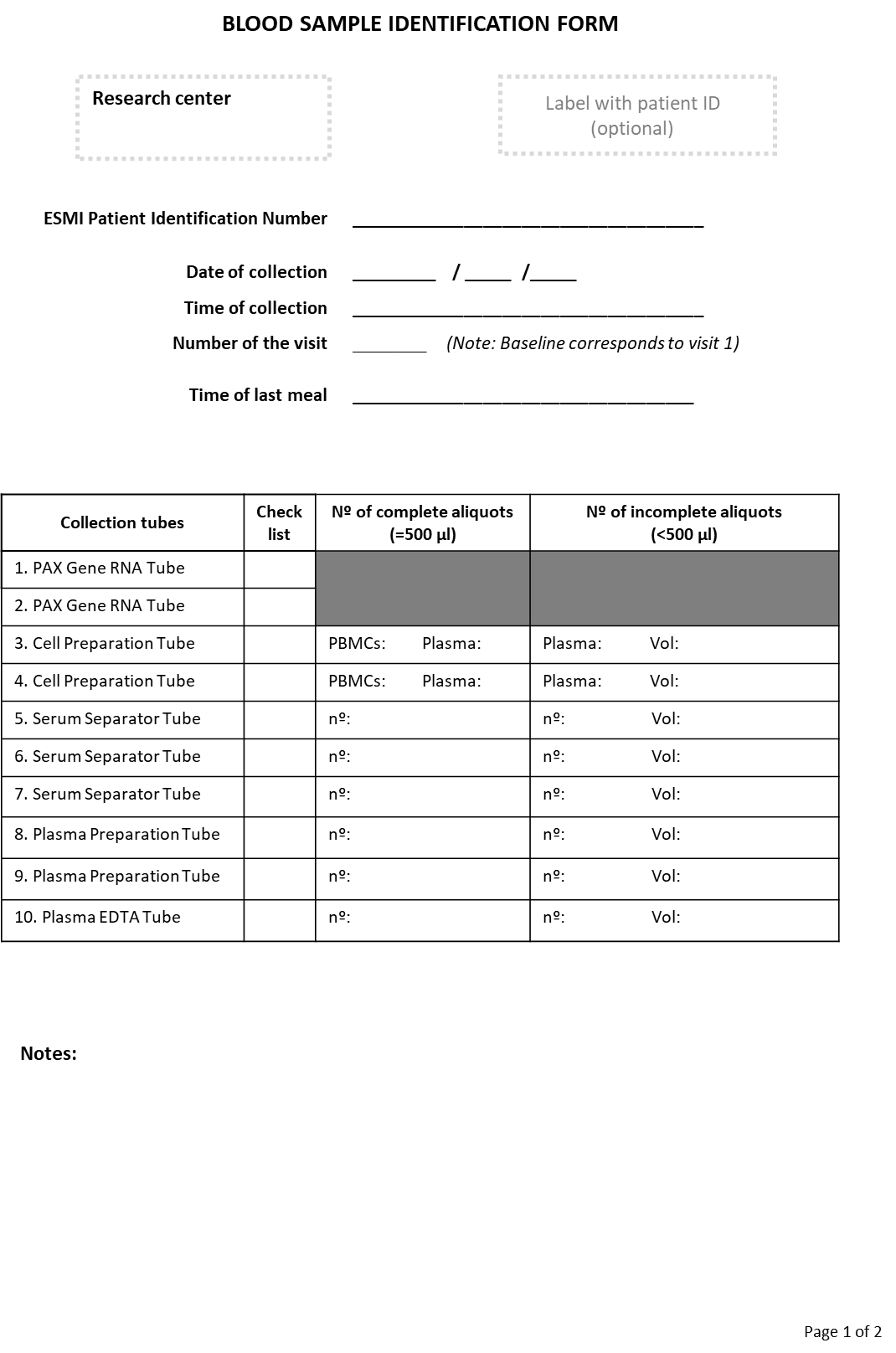
ANNEX IV – Blood identification form**


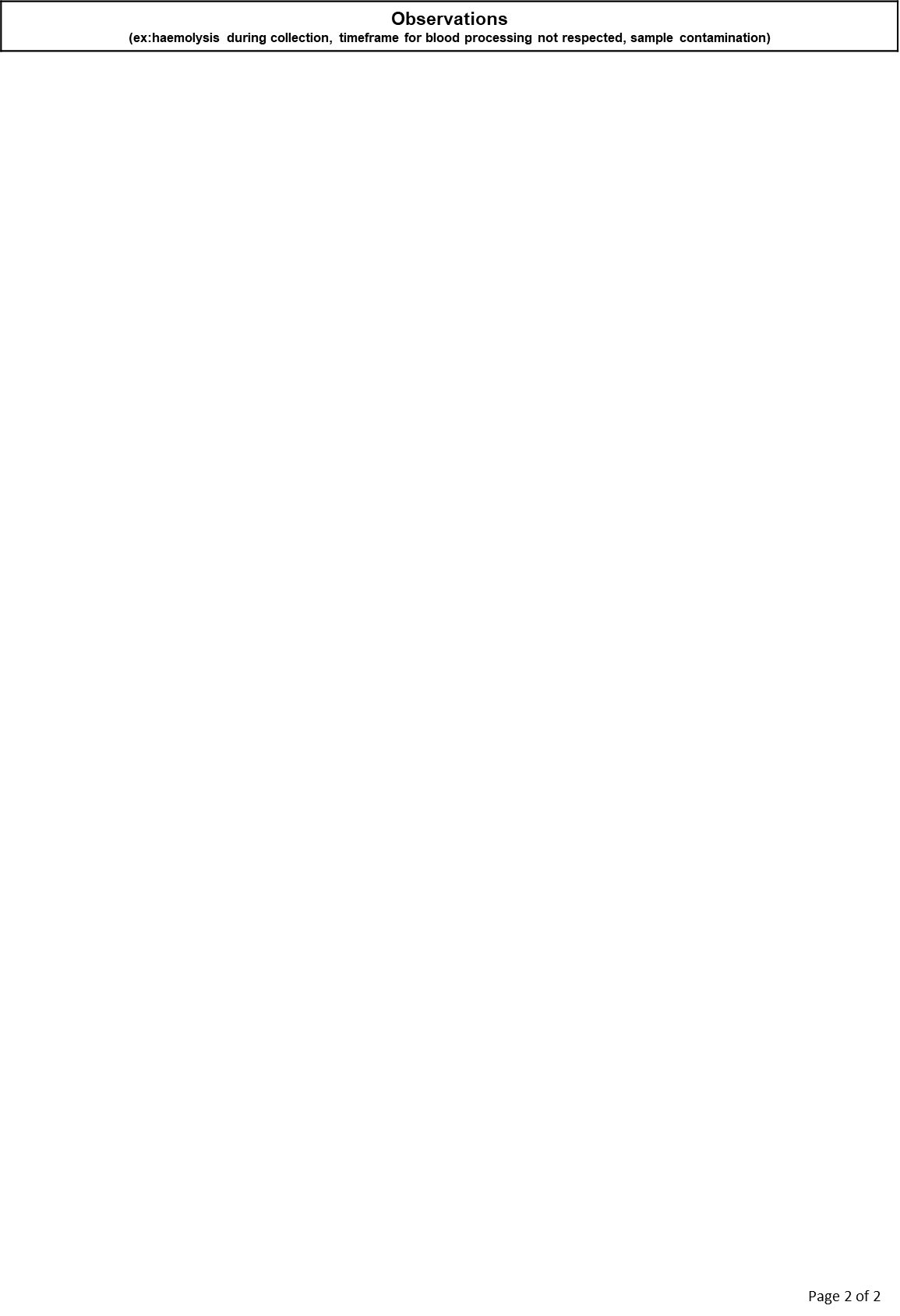


**
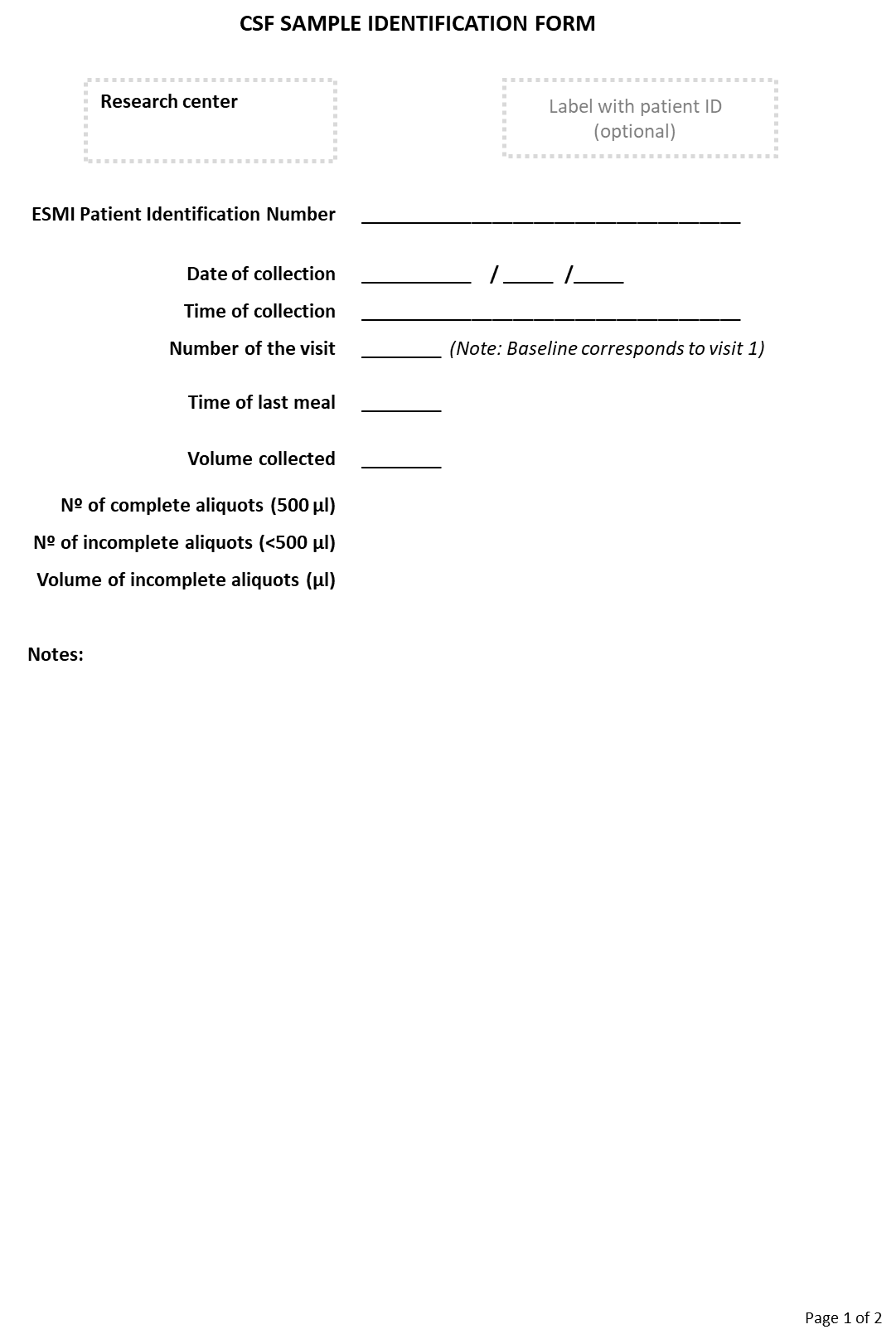
ANNEX V – CSF identification form**

**
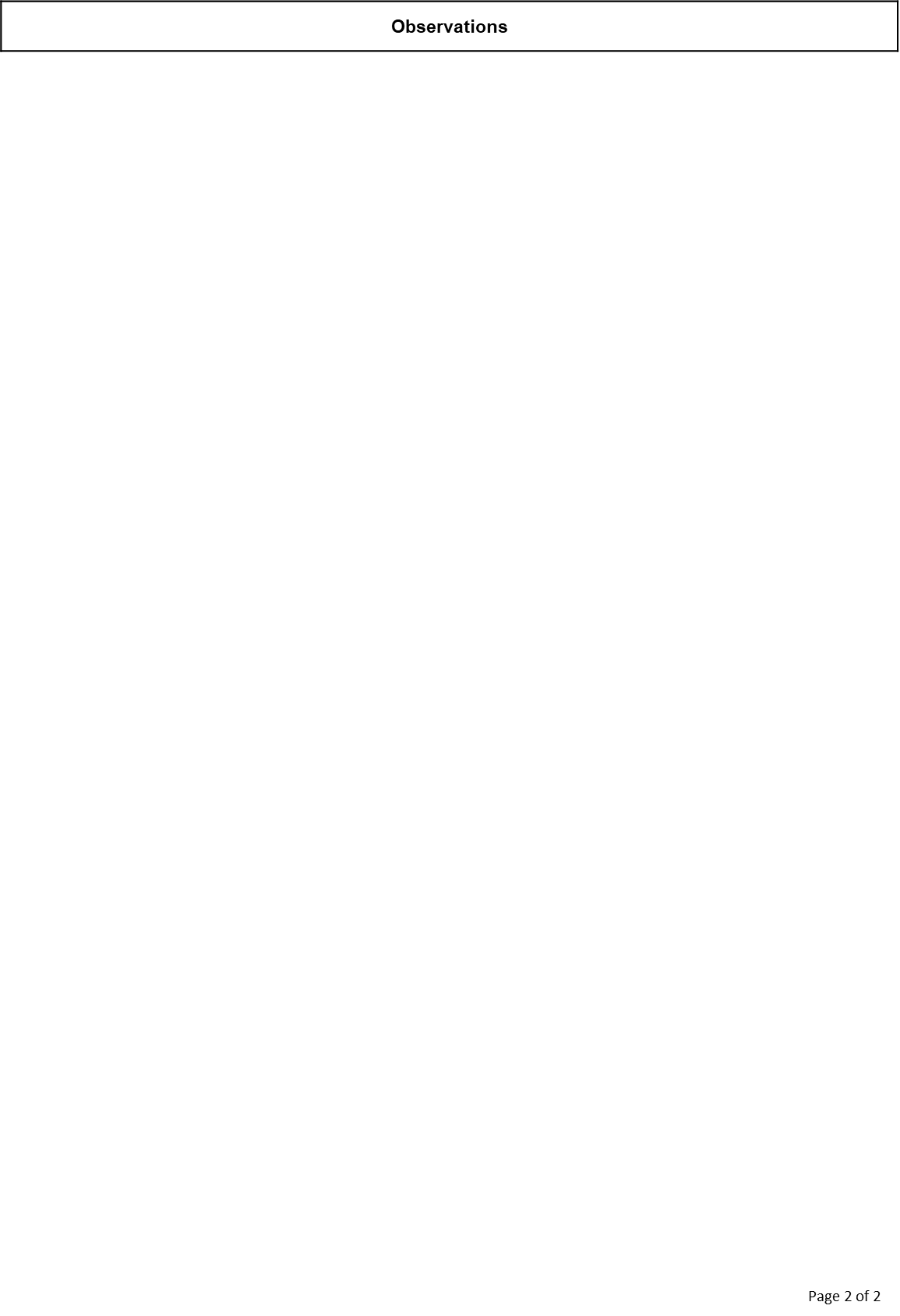
**

**ANNEX VI – Blood collection tubes per patient**

**#9**

**#4**

**#7**

**#2**

**#6**

**#5**

**#8**

**#10**

**#1**

**#3**


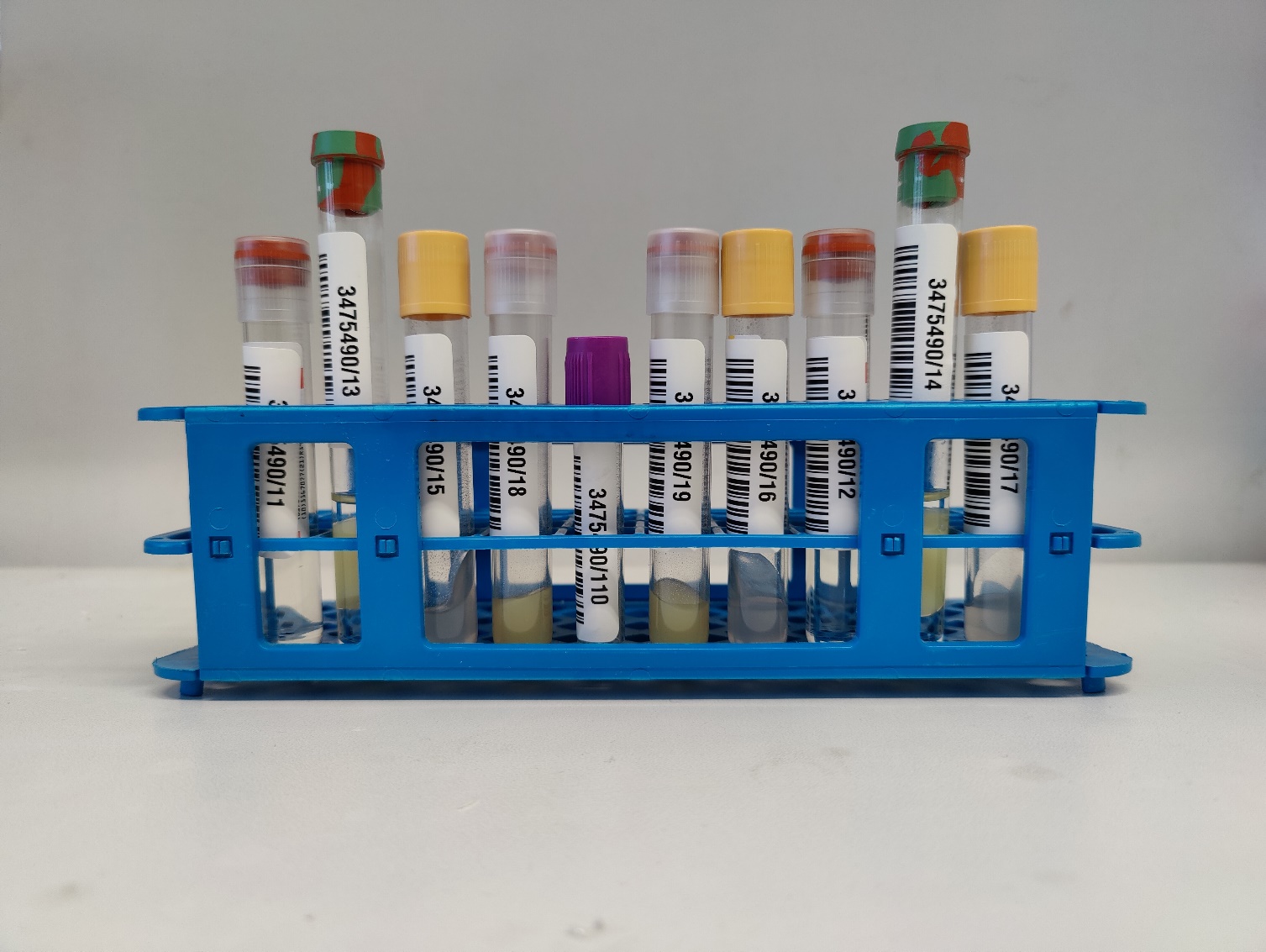


**ANNEX VII – Blood collection venipuncture procedure**


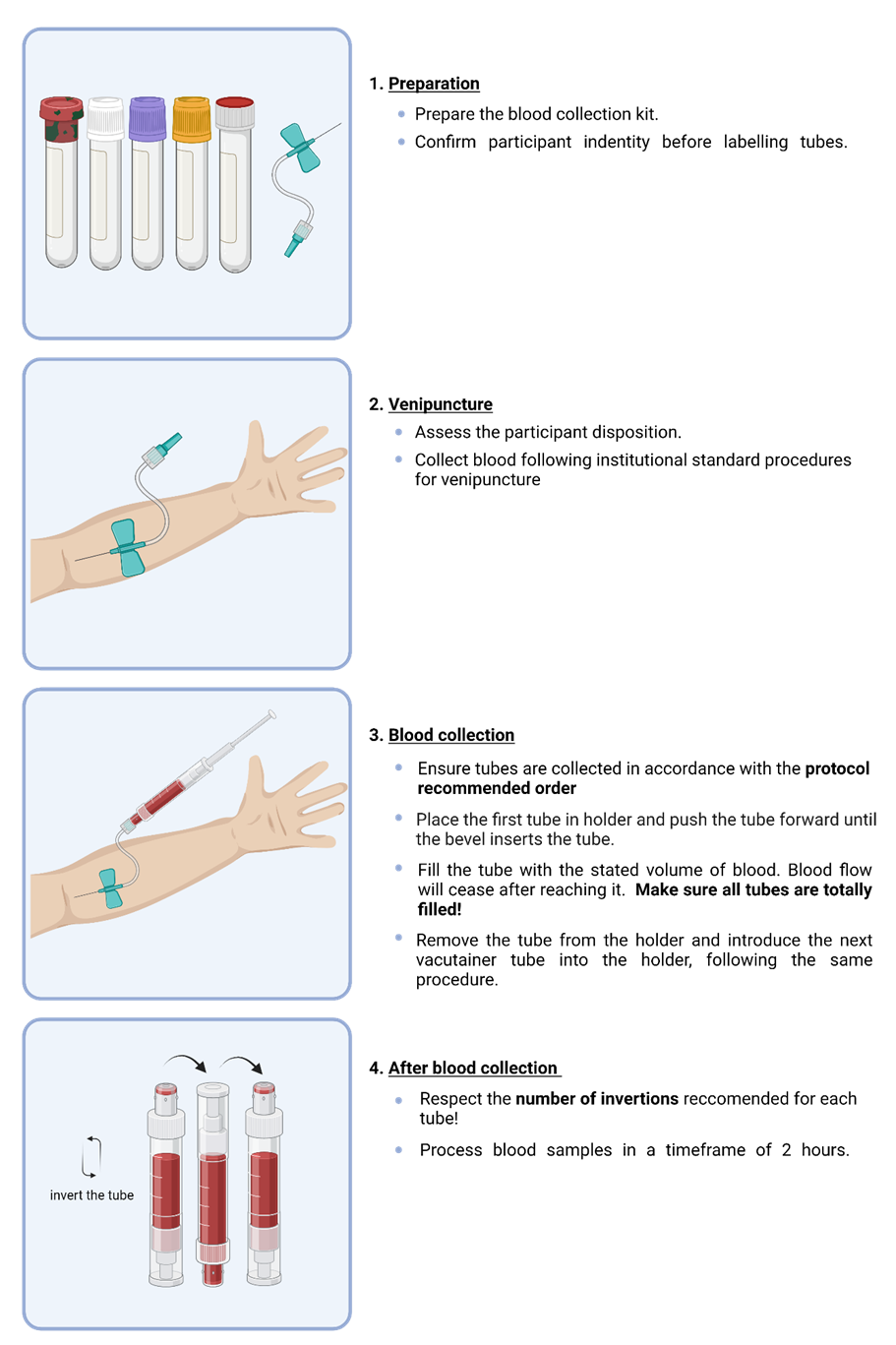


This figure was created with BioRender.com (agreement number: EA24YGCY0N).

**
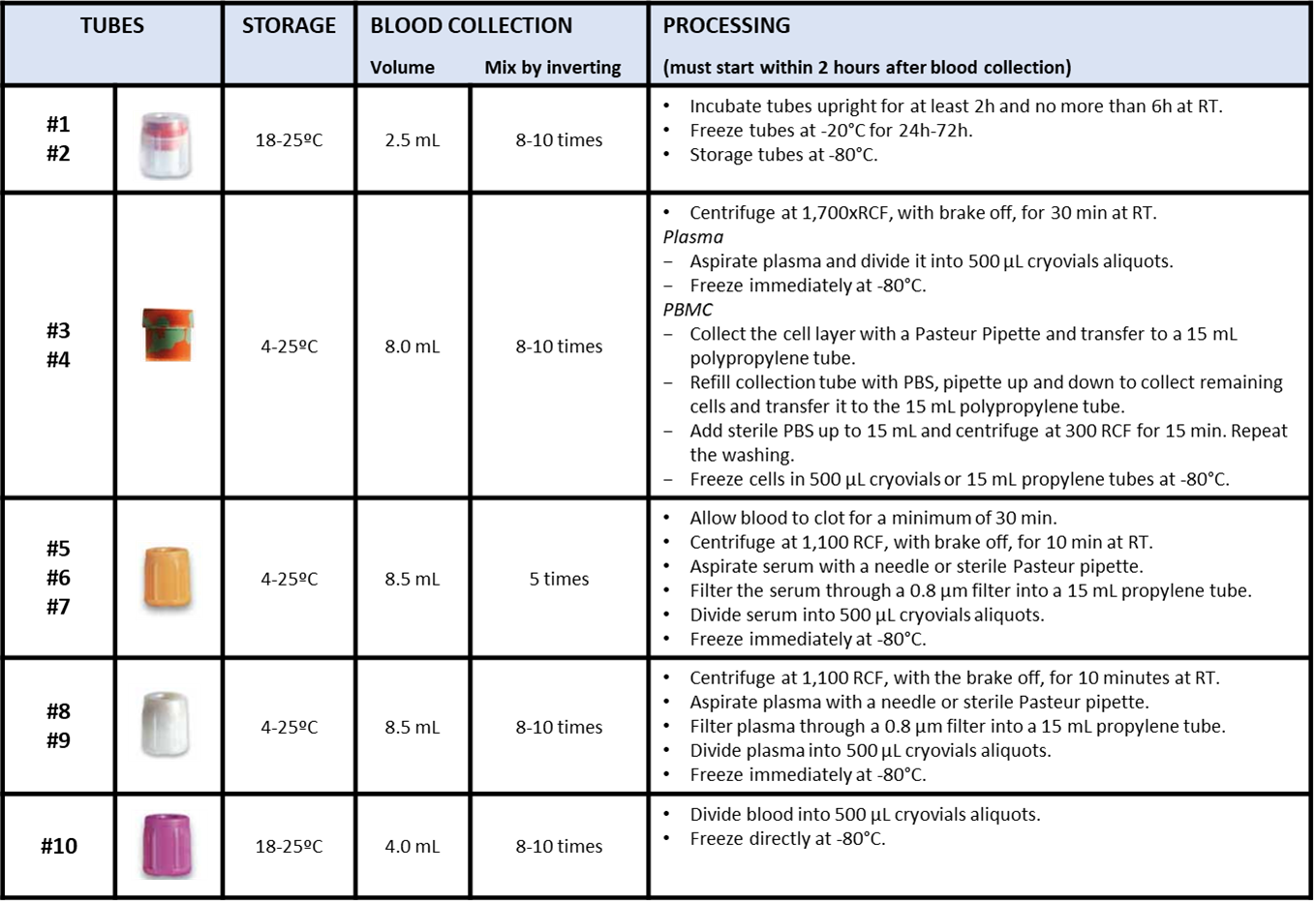
ANNEX VIII – Blood collection and processing**
